# 色谱分析实验教学革新与创新人才培养

**DOI:** 10.3724/SP.J.1123.2024.12019

**Published:** 2025-07-08

**Authors:** Jun HUANG, Zhanxia LYU, Zhen GAO, Meixian LI, Yinglin ZHOU

**Affiliations:** 北京大学化学与分子工程学院，北京 100871; College of Chemistry & Molecular Engineering，Peking University，Beijing 100871，China

**Keywords:** 色谱类实验, 教学革新, 科学思维, 自由探索, 创新人才培养, chromatography experiment, teaching reform, scientific thinking, free exploration, cultivation of innovative talents

## Abstract

色谱类实验是仪器分析实验的重要组成部分。为了适应新形势下培养具有科学思维创新人才的需求，自2017年起，北京大学化学与分子工程学院开设的仪器分析实验课程以色谱类实验教学为改革试点，通过加强实验内容设计，注重实际样品分析，提高学生参与度，增强学生自由探索度，强化学生分工合作并采用虚实结合的教学形式等一系列精心设计的改革举措，有效激发了学生学习的主观能动性，提升了学生对仪器分析原理和仪器结构功能的理解，培养了学生分析问题和解决问题的综合能力，强化了学生的科学思维和科学素养，有力促进了培养创新人才这一核心目标的实现。

## Abstract (en)

Chromatography experiments form an important component of an Instrumental Analysis Laboratory Course. In order to meet the demands of cultivating innovative talents with scientific thinking skills for the evolving educational objectives， the Instrumental Analysis Laboratory Course in the College of Chemistry and Molecular Engineering at Peking University has been reformed since 2017， with a focus on chromatography experiment teaching as a pilot project. A series of well-designed reform measures， including strengthening the design of the experimental content， focusing on the analysis of actual samples， increasing student participation， enhancing their freedom of exploration and the division of labor and cooperation， and adopting a teaching form with both virtual and real components， have been introduced. These measures have effectively stimulated students’ subjective learning initiatives， improved their understanding of the principles of instrumental analysis and the structures and functions of instruments， cultivated their abilities to comprehend， analyze， and solve problems， and strengthened their scientific thinking and literacy. Overall， the developed program has powerfully contributed to realizing the core goal of cultivating innovative talents.

分析化学是化学工作者依靠工具和自身感官来解剖和认识自然世界，并在不断总结和探索中诞生的重要的化学分支学科，对化学各分支学科的发展具有重要作用。欧洲化学联合会对分析化学定义如下：分析化学是发展和应用各种方法、仪器和策略以获得有关物质在空间和时间方面组成和性质的信息科学^［1，2］^。2018年国家自然科学基金委化学部对学科重新调整，新增化学测量学方向，旨在发展化学及相关学科的测量理论、原理、方法和技术，研制仪器、装置、软件及试剂，获取物质组成、结构、形貌、性质与功能等信息，揭示物质相互作用的分子基础和时空变化规律^［3］^。无论是欧洲化学联合会对分析化学的定义，还是国家自然科学基金委化学部对化学测量学发展目标的要求，都反映出分析化学涵盖了化学分析和仪器分析这两部分内容。仪器分析是在化学分析的基础上，伴随着物理学、电子技术和计算机技术的快速发展，通过使用仪器测量物质的物理或物理化学性质建立起来的各种分析方法，目前已成为分析化学的发展方向和学科发展的动力之一^［4］^。仪器分析作为现代化学工作者的第三只“眼睛”，在研究化学物质微观结构，发现新物质和创造新理论，促进学科的快速发展等方面发挥着越来越重要的作用。在化学类及与化学类相关专业中开设仪器分析课程的目的：一是让学生掌握化学研究中常用仪器分析方法的基本原理以及仪器的基本结构；二是通过对各类仪器分析方法的优缺点和适用范围的了解，使学生具备根据实验目的、样品性质以及实验成本等限制条件选择合适的仪器分析方法的能力^［5-8］^。

## 1 实验改革背景

在仪器分析实验教学中，首要且基础的目标是教导学生如何规范操作仪器，以降低失误操作的风险，防止在实验进程中损坏仪器。由于实际样品的分离分析往往耗时较长，若学生在未达标的情况下进行进样分析，难以在限定时间内收集到充足数据，进而影响实验结果的判断与分析。此外，学生普遍对分数较为看重，对实验失败抱有恐惧心理，导致在实验过程中过于谨慎，缺乏冒险精神。因此，在实验教学中，教师应积极鼓励学生勇于尝试，敢于用实验来验证自己的猜想，并允许在尝试中出现失误，使其在问题与失败中汲取经验、不断成长。教师不再将实验结果作为评判学生成绩的唯一标准，而是更加看重学生在实验报告中能否深入分析实验中存在的问题，并提出有效的解决方案或改进措施^［9，10］^。

仪器分析实验所使用的仪器较为昂贵且数量有限，损坏后没有替换设备，因此要求学生严格按照设定方案进行实验，以免仪器损坏影响整个实验教学进度；同时选课人数较多，常常是多人共用一台设备，严重影响学习效果，导致学生上课的积极性、参与度和主动性不高^［11］^。2016年北京大学全面启动教学改革，《仪器分析实验》课程由必修课改为专业限选课，同时为适应新形势下的学生特点，满足创新性拔尖人才培养的要求，仪器分析实验教学团队对课程内容和教学形式进行了全面梳理，选择仪器分析实验中占据重要地位的色谱类实验为切入点进行教学内容和教学模式改革，在教学实验中真实还原科研场景，关注科研中常见而教学中容易忽视的细节，培养学生从科研的视角思考问题，注重学生对理论知识的全面理解和应用，强化对学生科学思维和科研素养的培养^［12］^。下面详细介绍气相色谱、液相色谱、气相色谱-质谱联用实验改革措施以及虚实结合教学形式的开展情况。改革前后色谱类实验项目变化情况如表1所示。

**表1:** 色谱类实验项目改革前后对比表

Implementation time	Instrumental analysis method	Experimental content	Class hours	Satisfaction survey in 2024
Before 2016	GC	qualitative and quantitative analysis by GC	8	
	LC	quantitative analysis of PAHs in water samples by SPE and HPLC using the internal standard method	8	
	GC-MS	determining PAHs in the environment by GC-MS	4	
2017	GC	analyzing trace components in Chinese Baijiu by GC	6	80%
2018	LC	determining food pigments in beverages by LC	6	86.7%
2024	GC-MS	determining volatile components in coffee by headspace GC-MS	6	88.9%

GC： gas chromatography； LC： liquid chromatography； MS： mass spectrometry； SPE： solid-phase extraction； PAHs： polycyclic aromatic hydrocarbons.

## 2 气相色谱实验改革

改革之前气相色谱实验的分析对象是标准品或者用标准品配制的模拟样品，分析过程不存在杂质干扰，实验的主要目的是对色谱理论进行验证和训练学生的基础实验技能，让学生考察色谱实验中常见的流动相流速、柱温、固定相以及流动相等因素对样品分离的影响，这种培养模式对于夯实学生的基础理论知识和提高学生的实验操作水平具有十分重要的作用。但由于实验结果的可预测性，学生在实验中可发挥的空间有限，且相关内容易于在互联网上寻找，因此学生对于这类验证型实验的兴趣不大，参与感不强，学习效果不是十分理想。

2017年教学团队对气相色谱实验进行调整，第一次在色谱类实验中尝试分析实际样品即市售白酒，并允许学生自带感兴趣的白酒到实验课上分析。实验讲义中不提供具体的分离条件，要求学生通过自主设定升温程序和流动相流速，使白酒中所有微量组分在尽可能短的时间内实现基线分离。分离条件优化后，各组同学需学会用标准物质对照定性的方法合作完成对白酒中所有微量组分的定性，各组同学分工明确且独立操作，数据共享。白酒中甲醇的定量分析是通过内标法测定，学生需要了解内标法原理，通过配制适当浓度的甲醇和乙酸正丁酯（内标物）混合溶液，测定出甲醇相对于乙酸正丁酯的校正因子，最后通过在白酒中加入适量的乙酸正丁酯实现对白酒中甲醇含量的准确测定。

通过自行优化分离条件，能有效激发学生的主观能动性。学生需根据实验结果和所学知识不断调整实验参数，进一步理解柱温和流动相流速对气相色谱分离效果的影响，从而在运用所学知识解决实际问题过程中获得满满的成就感。这种密切联系生活的课题使学生在实验中的参与感更强，学生的兴趣和积极性也得到极大提高。某组学生在优化前后获得的白酒在气相色谱中的分离情况如图1所示。

**图1:**
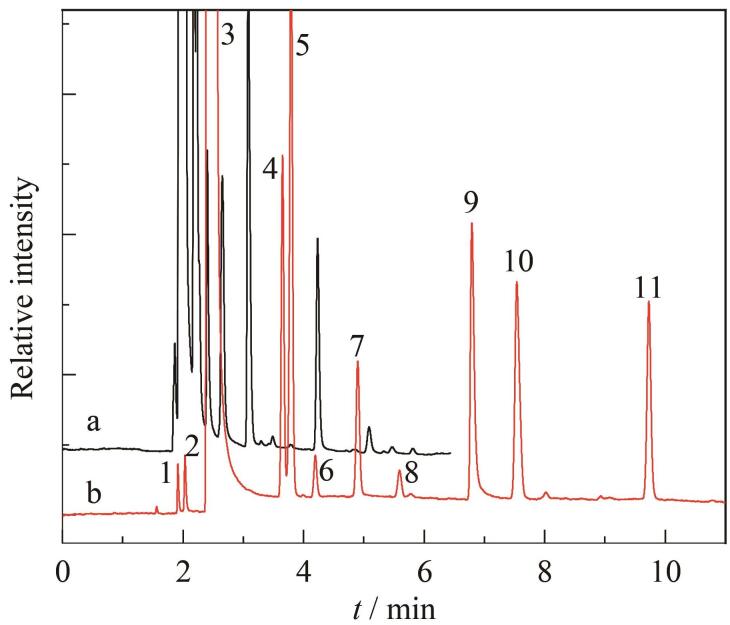
白酒在气相色谱中的分离情况

## 3 液相色谱实验改革

对过去10年本科毕业生的统计数据结果显示，北京大学化学学院超过80%的本科生毕业后将在国内或国外继续深造，这就意味着我们的教学场景应更贴近科研实践，让本科生更早在基础实验室开展真实的科研训练，成长为具有扎实实验技能和科学素养的专业人才，为其在相关研究领域取得成功提供坚实的科研思维支撑^［13，14］^。改革前的液相色谱实验中，待测样品是实验室技术人员配制：一类是含有尿嘧啶、萘、联苯和菲的甲醇混合溶液及其相应的单标溶液，供学生进行色谱柱参数的测定；另一类是含有萘、菲的被测水样，萘和菲的标准混合液以及内标物质，供学生进行固相萃取和内标定量。实验方案的设计注重学生操作技能的训练，缺乏学生自主探索的内容，样品前处理不会出现意外，学生按照步骤进行实验就可以取得相对满意的结果，这基本符合当时对人才培养的需要。然而新时代背景下，高等教育的目标是培养产生原创性成果的创新人才，要求学生善于思考、敢于实践。因此在实验内容设计上要给予学生自由探索的空间，要贴近真实科研环境。

2018年以来，HPLC实验开展了两方面改革，一方面允许学生自带感兴趣的饮料样品来实验室分析，样品分析对象有一定开放性；另一方面完全按照科研思维训练模式培养学生，增加学生自主探索的实验内容。具体的内容设计主要包括以下几方面。

（1） 选择相对复杂的实际样品，重视样品前处理

饮料样品为了满足口感、颜色、防腐等需要加入许多添加剂。将饮料样品作为实验对象，一方面有助于学生理解进入高效液相色谱中的样品应该具备何种条件，哪些可以直接进样，哪些需进行样品前处理；另一方面通过色谱图中杂质峰强度可以让学生了解样品前处理的好坏。此外，学生自带样品五花八门，需根据所带样品的性质对样品前处理方法进行调整，比如含有果肉的色素饮料，需先进行过滤或离心，取滤液或上清液进行吸附解吸实验。样品前处理常涉及废液回收，学生需分析哪些可回收，哪些可直接倒入下水道。这类实验既能提高学生的环保意识，又能减少废液的回收，降低实验室处理废液的经济成本。

（2） 增加等度与梯度洗脱方法对比，学会条件优化和理解泵压数据

通过等度洗脱与梯度洗脱方法对比，让学生在实验中真正了解两种洗脱模式的优缺点，并综合考虑实验需要、分离时间以及分离成本等因素，从而在实际操作中灵活选择合适的洗脱方法。在实验教学过程中，我们特别强调输液泵的泵压数据对液相色谱实验的重要作用，这一要求有助于培养学生记录泵压数据的良好习惯。在液相色谱的科研实践中，泵压数据作为一项至关重要的指标，能够揭示实验过程中的诸多关键信息，因此备受液相色谱科研工作者的密切关注。当实验中色谱分离效能下降与定性定量结果出现偏离时，往往可以先检查和分析泵压数据，进而推断流动相的成分或比例是否存在潜在的改变，此外泵压数据的波动还可预示整个色谱管路系统中是否存在泄露或堵塞的情况。学生通过对比梯度洗脱和等度洗脱的泵压情况，也可进一步理解影响泵压的常见因素，有助于学生从宏观和微观两方面理解两相平衡和样品组分在两相间的分配情况。总之，时刻关注并深入解析泵压数据，对于准确识别并有效解决实验过程中遇到的问题具有重要意义。

（3） 从实际科研角度出发，注重科学研究的逻辑性

在常规实验教学中，通常要求学生按照实验讲义配制外标法系列浓度，这缺乏真实科研的逻辑性和灵活性。在实际科研中，实际样品中的浓度并不可知，且不同样品或同一个样品的不同色素含量可能存在显著差异。因此，改革后学生自行根据定性时已知浓度色素的峰面积与样品中色素的峰面积估算样品中色素的浓度范围，再配制合适浓度的系列标准溶液。通过这种逻辑训练，学生能更好地理解和适应真实科研环境，提升独立解决问题的能力，为未来的科研工作打下坚实的基础。

（4） 注重色谱实验细节，加深对色谱理论的理解

标准试剂配制的模拟样品不存在杂质影响分离，学生做完实验，不需要在实验结束后冲洗色谱柱。而在实际样品分析时，经过样品前处理依然会有少量杂质不能完全除去。流动相中加入酸或缓冲盐的目的是通过调节pH改变化合物在色谱柱上的保留因子，从而达到改善峰形和提高分离度的目的。但流动相中添加酸或缓冲盐时，需要对色谱柱进行冲洗并将流动相切换成纯有机相，使色谱固定相浸润在有机相中。对于冲洗过程中色谱图背景基线和泵压发生的变化，可要求学生在实验课后专题分析变化原因。这样的教学安排，不仅有利于学生深入理解色谱理论，而且有利于他们关注实验中的细节问题，从而培养良好的实验习惯和科研素养，为其今后科研中获得高质量数据提供保障，表2统计了部分学生测定某款饮料中3种色素的含量结果。

**表2:** 学生测定的某款饮料中3种色素的平均含量

Pigment	Content/（μg/kg）	Number of students	RSD/%
Tartrazine	10.3	101	5.5
Carmine	2.4	89	8.8
Sunset yellow	14.4	95	5.3

液相色谱实验结束后冲洗过程的背景基线变化情况如图2所示。从a点开始将其中的醋酸铵流动相换成水，背景基线有小幅降低。从b点开始将水换成甲醇后，背景基线逐渐升高，随后降低并保持不变，这是因为甲醇的紫外吸收比水高，背景基线逐渐升高。图中的小峰是经过前处理的样品中含有的杂质峰，其在分离条件下保留在柱子上，当流动相变为100%的甲醇时，流动相的极性下降，杂质被洗脱下来。当杂质全部冲洗下来后，c点之后的背景基线保持不变，但均高于a点和b点的背景基线。

**图2:**
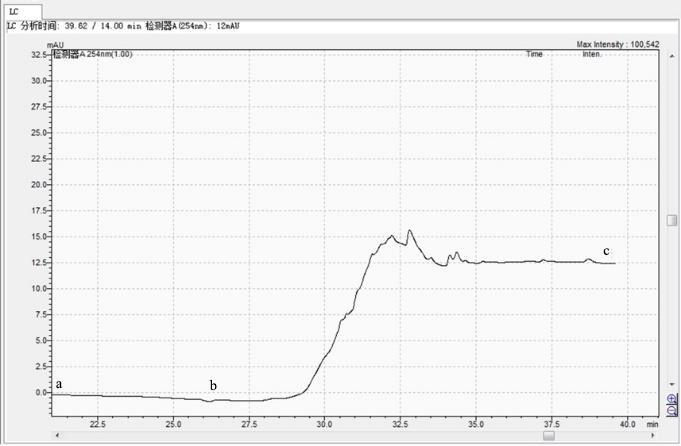
液相色谱实验结束后冲洗时基线变化图

## 4 气相色谱-质谱实验改革

2024年的GC-MS实验引入了顶空装置，以师生们普遍喜爱的咖啡作为分析对象，学生亲自研磨咖啡豆并制作分析样品，用顶空气相色谱-质谱联用装置测定咖啡粉中挥发性成分。静态顶空GC-MS联用测定咖啡中挥发性物质流程示意图如图3所示。实验中学生要设定的参数比较多，除了要设定程序升温参数，还需设定质谱参数及顶空进样参数，对于第一次接触顶空气相色谱-质谱仪的学生来说，出错是难免的。为了使学生深刻理解每个参数的含义，必须通过引导学生自主调整参数，并观察这些调整对分离效果所产生的影响。如果参数设置不当，虽不能获得理想分离分析效果，但对于学生认识参数的作用和意义十分有用。另外，实验室提供极性和弱极性固定相两种色谱柱，组和组之间的同学通过共享数据，对比在不同极性色谱柱上咖啡挥发性组分的分离情况和出峰顺序（如图4所示），进一步理解色谱分离原理，掌握优化分离的方法，并学会用质谱鉴定咖啡中的组分。

**图3:**
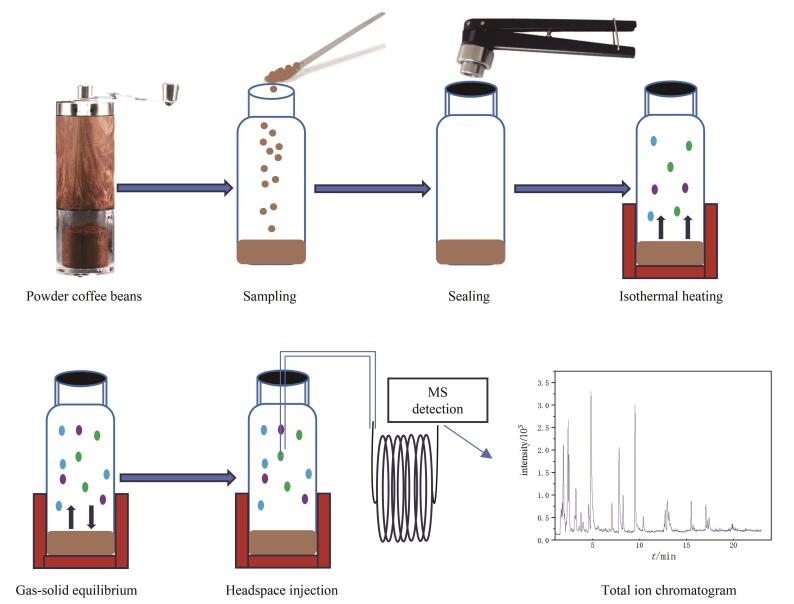
静态顶空气相色谱-质谱联用测定咖啡中挥发性物质的流程示意图

**图4:**
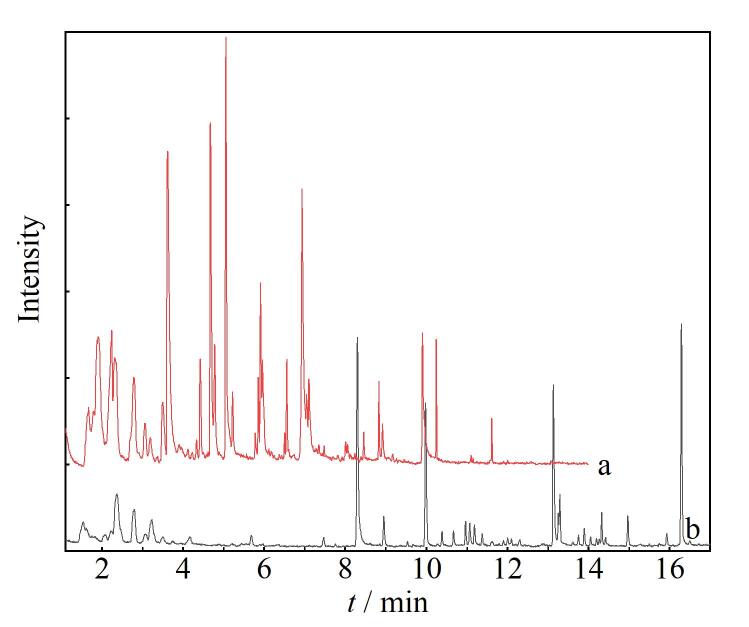
不同极性色谱柱对咖啡挥发性成分的分离效果对比

## 5 虚实结合的教学形式

仪器分析学习之所以具有挑战性，主要在于涉及多个学科知识体系，且仪器内部元件因稳定性和集成的需要，常包裹在盒子里，就像一个“黑匣子”，外部看似一样，但内部结构千变万化。特别是色谱类仪器，不仅检测器种类多，分离模式多，进样方式也不尽相同。为了帮助学生洞察仪器运行原理、内部构成及组件功能，我们采用虚实结合的教学模式，一方面拍摄仪器分析实验慕课，供学生在实验课前预习，使其对仪器结构和功能有初步了解；另一方面，针对那些不易理解的知识和内容，精心制作动画视频，比如色谱中六通阀进样过程、往复式机械泵运行原理、流动相自动脱气原理、分流/不分流进样原理、液相色谱中紫外检测器Z字型检测原理、质谱中化合物离子化及四极杆分离过程等，加深学生对这些原理的理解；此外，我们还收集学院各课题组淘汰的仪器设备，打开“黑匣子”的盖子，拆解部分零部件，让学生直观地观察仪器的内部结构。通过不同时期的仪器设备对比，学生可在一定程度上了解仪器的发展脉络和新仪器的改进和升级，进而激发学生对仪器分析未来发展的好奇心和探知欲。所拆解的淘汰设备的内部结构和零部件见图5。

**图5:**
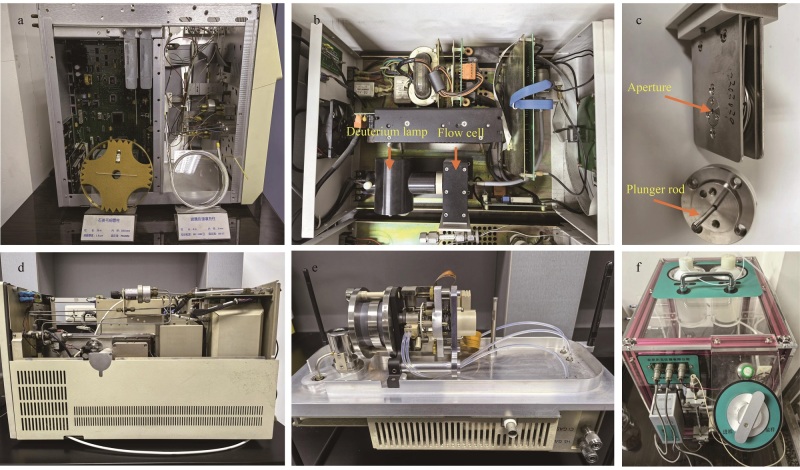
拆解的淘汰设备的内部结构和零部件图

## 6 结语

经过上述改革措施的实施，学生对色谱类实验教学的满意度得到显著提升，课堂上学生的参与度、积极性以及与授课老师的互动性都得到明显提高；学生不仅深刻理解了色谱类仪器的分离原理、结构和功能，还初步掌握了根据样品信息和任务目标来选择合适的色谱类分析方法，并能对实验方案进行有针对性的设计和探究。此外，学生在实验中更加注重分工合作和数据共享，在实验过程积极交流并共同解决实验中遇到的问题，能深切感受到所学知识的实用性以及实验带来的快乐。通过这些积极的改革举措，限选课人数逐年增加，2018年春季为40人，2018年秋季增加到77人，2019年秋季上升为117人，2020-2023年稳定在120人左右。尽管教学改革取得令人满意的阶段性效果，但我们也清醒认识到，改革后的实验内容具有一定的开放性，学生课内自主性的增加给授课老师在教学环节以及实验支撑岗位技术人员在仪器设备维护维修方面都提出了更大的挑战，尤其是学生自带样品和自主设计实验方案所带来的不确定性。今后我们将继续秉持培养学生的科学思维和科学素养目标，进一步完善实验教学内容，探索新的教学模式，特别是人工智能（AI）技术的蓬勃发展，对教学改革提出新的要求，需进一步转变思路，让AI赋能仪器分析实验教学。我们期待，围绕色谱实验持续开展的教学改革措施能够对于其他高等院校化学类拔尖人才培养提供有益的借鉴、思路和方向。
